# Linking Short-Chain Fatty Acids to Systemic Homeostasis: Mechanisms, Therapeutic Potential, and Future Directions

**DOI:** 10.1155/jnme/8870958

**Published:** 2025-07-28

**Authors:** Yueru Zhao, Jing Chen, Yunlong Qin, Jinguo Yuan, Zixian Yu, Rui Ma, Fude Liu, Jin Zhao

**Affiliations:** ^1^School of Clinical Medicine, Health Science Center, Xi'an Jiaotong University, Xi'an, Shaanxi, China; ^2^Department of Nephrology, Xijing Hospital, Fourth Military Medical University, Xi'an, Shaanxi, China; ^3^Department of Neurosurgery, Tangdu Hospital, Fourth Military Medical University, Xi'an, Shaanxi, China; ^4^Department of Geriatrics, Xijing Hospital, Fourth Military Medical University, Xi'an, Shaanxi, China; ^5^Department of Neurology, The First Affiliated Hospital of Xi'an Jiaotong University, Xi'an, Shaanxi, China

**Keywords:** gut microbiota, histone deacetylases inhibitor, immune homeostasis, short-chain fatty acids, tumorigenesis

## Abstract

Short-chain fatty acids (SCFAs), pivotal metabolites derived from microbial fermentation of dietary fiber, serve as critical modulators of glucose and lipid metabolism. Dysregulation of SCFA levels, often stemming from inadequate fiber intake or dysbiosis of SCFA-producing microbiota, correlates with heightened susceptibility to diverse pathologies, including autoimmune disorders, metabolic syndromes, and malignancies. Emerging evidence underscores the pleiotropic roles of SCFAs in orchestrating gut and systemic homeostasis, positioning them as novel therapeutic candidates for immune dysregulation, inflammatory conditions, and transplant rejection. This review synthesizes current knowledge on SCFA biosynthesis, absorption dynamics, and their multifaceted regulatory mechanisms, spanning epigenetic modulation, G protein–coupled receptor (GPR) signaling, and immune cell crosstalk. We further elucidate their therapeutic potential in clinical contexts, emphasizing their capacity to recalibrate immune responses, suppress chronic inflammation, and mitigate oncogenesis. By integrating recent advances in microbiome research and translational applications, this work highlights the imperative for precision interventions targeting SCFA pathways to bridge the gap between microbial ecology and clinical innovation.

## 1. Introduction

The gastrointestinal tract represents a dynamic ecosystem where host cells, immune mediators, and microbial metabolites collaboratively maintain equilibrium. Perturbations in gut homeostasis are implicated in the pathogenesis of diverse conditions, including inflammatory bowel disease (IBD) [1], obesity [2], diabetes [3], colorectal cancer (CRC) [4], coronary heart disease [5], kidney diseases [6, 7], and neuropsychiatric conditions [8, 9]. Among microbial metabolites, SCFAs, primarily acetate, propionate, and butyrate, have emerged as keystone molecules linking dietary patterns, microbiota composition, and host physiology. Synthesized through anaerobic fermentation of indigestible polysaccharides, SCFAs exert systemic effects via receptor-mediated signaling, epigenetic regulation, and metabolic reprogramming [10–12].

Despite promising preclinical data, clinical translation remains hindered by incomplete mechanistic insights and interindividual variability in intervention outcomes. Current strategies, such as SCFA enemas [13] or oral supplementation [14], demonstrate partial efficacy, necessitating a deeper understanding of context-dependent SCFA actions. Additionally, the multifaceted regulatory effects exerted by dietary fiber (DF)–derived SCFAs as biological mediators may also underscore their promising preventive potential in mitigating numerous prevalent diseases through simple and cost-effective dietary intervention. This review advances the discourse by delineating tissue-specific SCFA effects, dissecting their immune-metabolic interplay, and proposing targeted therapeutic frameworks. By synthesizing cutting-edge research, we aim to catalyze the development of SCFA-centric therapies tailored to individual microbiome profiles and disease states.

## 2. Biosynthesis and Bioavailability of SCFAs

### 2.1. Microbial Pathways of SCFA Production

SCFA biosynthesis is intricately linked to microbial substrate utilization. Resistant starch and amino acids serve as primary precursors, with cysteine, alanine, serine, glutamate, and basic amino acids (lysine, histidine) contributing to acetate and butyrate synthesis via cross-feeding pathways [15, 16]. Pyruvate conversion via the Wood–Ljungdahl pathway or acetyl-CoA synthetase is predominant in Bacteroidetes and Firmicutes [17]. Cross-feeding mechanisms involve the conversion of acetate to butyrate by Faecalibacterium prausnitzii and Roseburia spp., mediated by the enzyme butyryl-CoA:acetate CoA-transferase [18, 19]. Specialized species, such as *Eubacterium rectale*, *Eubacterium ramulus*, and *Coprococcus-related strain L2-50*, have the capacity to directly utilize glucose for butyrate formation owing to the existence of phosphotransferase and butyrate-kinase [18, 20]. In addition, certain bacteria belonging to Firmicutes, Fusobacteriales, and Bacteroidetes employ amino acids to yield butyrate via the lysine, glutarate, and 4-aminobutyrate metabolic pathways [21]. Propionate synthesis is mediated through succinate, acrylate, and propanediol pathways, with taxonomic specificity. The acrylate pathway, found only in a limited number of Negativicutes and Lachnospiraceae, utilizes lactate to enhance propionate production. Additionally, deoxy sugars, like fucose and Rhamnose, serve as precursors for propionate formation in Lachnospiraceae species, *Ruminococcus obeum*, and *Roseburia inulinivorans* through the propanediol pathway [22]. Unlike acetate, propionate, and butyrate, primarily from carbohydrate fermentation, branched-chain fatty acids (BCFAs), such as isobutyrate and isovalerate, originate from microbial proteolysis of valine and leucine [23]. Valerate arises via both carbohydrate by *Megasphaera spp.* and amino acid pathways by *Clostridium spp.* [24–26]. BCFAs are biomarkers of protein fermentation, accumulating under high-protein/low-fiber diets or dysbiotic conditions [27, 28].

Environmental factors, including pH, nutrient availability, and redox potential, modulate microbial community structure and SCFA output [29, 30]. For instance, iron limitation enhances butyrate production by favoring obligate anaerobes, while peptide abundance shifts metabolic flux toward proteolytic SCFA generation, suggesting environmental adaptability in SCFA synthesis [30].

### 2.2. Absorption and Systemic Distribution

**Figure 1:**
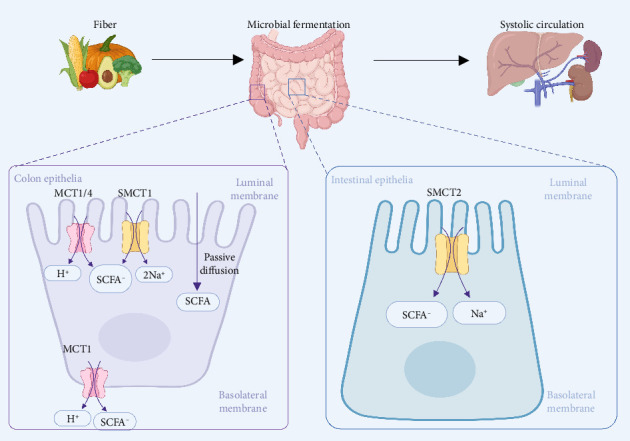
Absorption and systemic distribution of SCFAs. Dietary fiber undergoes microbial fermentation to produce SCFAs, which are absorbed by colon and intestinal epithelia via MCT1/4 and SMCT1/2 transporters. H^+^ gradients and Na^+^ symport mechanisms facilitate cellular uptake and systemic dissemination, enabling SCFAs to exert pleiotropic effects across tissues. SCFAs, short-chain fatty acids; MCT, monocarboxylate transporters; SMCT, sodium-coupled monocarboxylate transporter.

Given that the pH of the colon ranges from 5.5 to 6.7, most SCFAs exist as ions in the intestinal tract, with only a small fraction of undissociated SCFAs passively diffusing into epithelial cells [31]. Approximately 90%–95% of SCFAs are absorbed in the colon and cecum, primarily via monocarboxylate transporter 1 (MCT1/SLC16A1), sodium-coupled monocarboxylate transporter 1 (SMCT1/SLC5A8), and passive diffusion [32]. Regional concentration gradients (70–140 mM proximally vs. 20–70 mM distally) reflect differential epithelial absorption rates and microbial activity [33]. MCT1 demonstrates broad apical and basolateral membrane localization throughout colonic epithelial cells, whereas SMCT1 and MCT4 (SLC16A3) show exclusive apical and basolateral membrane expression, respectively [34]. These three transporters display tripartite affinity for acetate, propionate, and butyrate with differential transport kinetics [32]. In contrast, the low-affinity transporter SMCT2 (SLC5A12), predominantly expressed in the small intestine, specifically mediates the absorption of diet-derived SCFAs rather than those produced through bacterial fermentation [34, 35]. The simultaneous transport of SCFAs through the transporters is accompanied by the absorption of sodium and chloride ions and the secretion of bicarbonate, thereby maintaining intestinal electrolyte balance [34]. Postabsorption, hepatic first-pass metabolism limits systemic butyrate and propionate to micromolar levels, whereas acetate circulates widely, due to peripheral tissue utilization [36] (Figure 1).

## 3. SCFAs in Immune Equilibrium

### 3.1. Antibody Response and B-Cell Regulation

Butyrate, via suppressing Histone deacetylase (HDAC) activity, transcriptionally regulates critical B-cell functional genes, such as *Aicda*, *Xbp1*, and *Prdm1*, leading to inhibition of class-switch recombination (CSR), somatic hypermutation (SHM), and plasma cell differentiation [37]. Notably, propionate exhibits dose-associated regulatory effects on CSR processes. Low concentration of propionate moderately enhances CSR and SHM through homeostatic immunomodulation, while elevated SCFA levels within a broad physiological range demonstrate therapeutic potential in autoimmune conditions, as evidenced by their immunosuppressive effects in murine lupus models [38, 39]. In contrast, acetate demonstrates distinct immunoregulatory properties by enhancing IgA specificity through CD4^+^ T-cell interactions while simultaneously promoting T-cell-independent IgA production via GPR43 signaling [40, 41].

SCFAs collectively orchestrate immunoglobulin production through dendritic cell (DC)–mediated pathways. These microbial metabolites stimulate B-cell activating factor secretion and enhance ALDH1a2 expression, thereby facilitating IgA and IgG synthesis in B lymphocytes [42]. Oral administration of SCFAs and DF significantly elevated cecal IgA and serum IgG levels through energy metabolism of plasma cells. This metabolic enhancement involves multiple pathways: increased acetyl-CoA production, elevated fatty acid synthesis, glycolytic pathway activation, and augmented mitochondrial oxidative phosphorylation [43].

In clinical applications, *Clostridium butyricum*–derived butyrate demonstrated therapeutic efficacy in allergic disorders through HDAC1 inhibition, particularly enhancing given regulatory B-cell (Bregs) populations and interleukin-10 (IL-10) expression during specific immunotherapy [44, 45]. Mechanistically, butyrate activates the mammalian target of rapamycin (mTOR) pathway in Bregs, promoting IL-10 secretion [46]. Furthermore, it potentiates Breg functionality through upregulation of 5-hydroxyindole-3-acetic acid (5-HIAA), a serotonin-derived aryl hydrocarbon receptor (AhR) agonist. This cascade ultimately suppresses germinal center B-cell proliferation and inhibits terminal plasma cell differentiation, establishing butyrate as a critical immune-metabolic regulator in both physiological and pathological immune responses [47].

### 3.2. T-Cell Polarization and Plasticity

The regulatory effects of SCFAs on Treg cells and effector T cells are highly complex, involving cytokine environment, immune status, and dependent on the HDI activity [48]. Butyrate induces extrathymic Treg differentiation via FOXP3 acetylation, whereas acetate and propionate enhance colonic Treg accumulation through GPR43 signaling [49, 50]. Research by Martin-Gallausiaux et al. demonstrated that butyrate upregulates the SP1 signaling pathway and amplifies transforming growth factor-β (TGF-β) expression in intestinal epithelial cells, thereby driving localized Treg accumulation in the gut [51]. Furthermore, under immune homeostasis, SCFAs activate the mTOR–S6K pathway to promote the differentiation of naïve T cells into IL-10^+^ Treg [48, 52]. Intriguingly, the butyrate receptor, GPR109A, expressed on macrophages and DCs, indirectly facilitates the Foxp3^+^ and IL-10^+^ Treg differentiation [53]. Valerate enhances IL-10 production and suppresses IL-17 expression by improving lymphocyte metabolism and modulating their epigenetic status, respectively [54].

During active immune responses, however, acetate and propionate remarkably support the polarization of naïve T cells into T helper (Th) subsets, such as Th1 and Th17 cells, and regulate interferon-γ (IFN-γ), IL-10, and IL-17 secretion via STAT3 and mTOR pathway [48]. SCFAs exhibit divergent regulatory effects on Th1 and Th17 differentiation. Butyrate positively influences Th1 development, promoting IFN-γ and T-bet expression during cell differentiation. Conversely, in the case of Th17 polarization, the differentiation and development of Th17 are suppressed by inhibiting IL-17, retinoic acid–related orphan receptor α (RoRα), and RoRγt [55]. Sun et al. [56] revealed SCFAs inhibit excessive Th1 activation and induce IL-10 production by the STAT3/mTOR axis. This mechanism correlates with the high GPR43 expression on Th1 cells, whereas naïve T cells exhibit limited responsiveness due to receptor scarcity [48, 55]. Similarly, butyrate restrains hyperactive immune responses during Th17 differentiation by inducing Blimp-1 and IL-10 [55]. Notably, Blimp-1 has demonstrated therapeutic potential in autoimmune disease models by modulating Th1/Th17 differentiation and Treg function, suggesting that SCFA-mediated Blimp-1 activation may offer novel immunosuppressive therapeutic strategies [57].

Additionally, SCFAs critically regulate the Th9/IL-9 axis. In dextran sulfate sodium (DSS)–induced colitis model, anti-IL-9 antibody administration alleviated disease severity [58]. Butyrate and propionate selectively upregulate *FOXP3* gene expression while inhibiting Th9 differentiation and infiltration in ovalbumin-induced pneumonia. Conversely, adoptive transfer of Th9 cells or exogenous IL-9 exacerbated eosinophil infiltration and counteracted SCFA-mediated anti-inflammatory effects, confirming that SCFAs mitigate excessive inflammation by targeting the Th9/IL-9 [59]. Collectively, these findings elucidate the multidimensional regulatory network through which SCFAs govern T-cell fate, providing a theoretical foundation for precision immunotherapy in immune-related disorders.

### 3.3. Mononuclear Phagocyte Regulation

DF and its microbial fermentation–derived SCFAs modulate mononuclear phagocyte activity through multifaceted pathways critical for immune homeostasis. A high-fiber diet promotes the differentiation of bone marrow-derived Ly6C-patrolling monocytes, thereby suppressing neutrophil-driven hyperinflammatory responses against viral infections. Concurrently, SCFAs enhance CD8^+^ T-cell metabolism and antiviral functionality via epigenetic [60]. Mechanistically, SCFAs regulate cytokine dynamics in human peripheral blood mononuclear cells (PBMCs) by suppressing monocyte chemotactic protein-1 (MCP-1), tumor necrosis factor-α (TNF-α), and IFN-γ, while modulating prostaglandin E2 (PGE2) and IL-10 production [61–63]. Notably, dose- and time-dependent effects are evident, whereby elevated SCFA concentrations (> 2 mM) induce proinflammatory cytokines (IL-8, IL-6, IL-1β, TNF-α) in PBMCs and neutrophils through Toll-like receptor (TLR)–mediated signaling [64].

SCFAs exhibit remarkable adaptability in macrophage regulation, tailored to local or systemic immune contexts. In immunocompromised hosts with *Klebsiella pneumoniae* pneumonia, SCFA supplementation enhances macrophage phagocytic capacity by targeting LAMTOR2, a key effector of phagosome–lysosome fusion, and activating extracellular signal-regulated kinase (ERK) signaling [65, 66]. Butyrate preferentially drives monocyte differentiation into macrophages with heightened antibacterial activity, coupled with glycolysis suppression and mTOR inhibition via HDI [67]. However, in murine models of Type 1 diabetes (T1D), acetate activates GPR43, triggering ERK-dependent apoptosis of infiltrating macrophages to resolve inflammation and improve glucose homeostasis [68]. Atherosclerotic plaque stability is further influenced by butyrate, which reduces macrophage adhesion and migration via downregulation of CD36, proinflammatory factors, nuclear factor κb (NF-κB), and steroid receptor coactivator (Src) activity [69–71]. Additionally, butyrate reprograms IL-4-induced M2-like macrophages into highly phagocytic phenotypes with diminished proinflammatory cytokine secretion [72].

The anti-inflammatory properties of SCFAs are mediated through distinct molecular pathways [72]. Butyrate suppresses NF-κB signaling pathway in macrophages, reducing nitric oxide (NO), TNF-α, and IL-12 production independently of IL-10, presenting a potential ecological approach for the management of colitis [73, 74]. GPR41/43 mediates the inhibitory effects of acetate on IL-6 and IL-8, as well as propionate or butyrate on IL-6 production, while HDI activity underlies their suppression of vascular adhesion molecules and PBMC adhesion, mitigating atherosclerosis risk [75–77]. In addition, butyrate upregulates IL-10 and IL-18 secretion by intestinal epithelial cells, macrophages, and DCs through GPR109A signaling, reinforcing epithelial integrity, and dampening inflammation [78]. Similarly, acetate from *Escherichia coli* KUB-36 downregulates IL-1β, IL-6, IL-8, and TNF-α, while elevating anti-inflammatory IL-10 [79]. These findings underscore SCFAs as versatile immunomodulators with context-dependent roles in balancing inflammatory responses and maintaining immune equilibrium.

### 3.4. Granulocyte Modulation

Persistent neutrophil infiltration during chronic inflammation significantly exacerbates barrier dysfunction and tissue damage [80, 81]. SCFAs exert early-stage regulatory effects on monocytes, effectively attenuating neutrophil recruitment, a mechanism validated across multiple inflammatory models [60, 82]. DF deficiency disrupts gut microbiota homeostasis, elevating chemokine ligands (CXCL) 1 and CXCL2 on neutrophil surfaces, thereby enhancing susceptibility to inflammatory triggers such as DSS [82].

SCFAs demonstrate stage-specific modulation of neutrophil activity, reflecting their dual role in resolving acute infections and mitigating chronic inflammation. In GPR43-deficient mice, short-term lipopolysaccharide (LPS) stimulation (1 h) elicited a surge in the rolling and adhesion of neutrophils, whereas prolonged LPS exposure (4 h) reduced neutrophil velocity and amplified vascular accumulation, indicative of SCFA-dependent regulation of infection dynamics [83, 84]. Furthermore, SCFAs prolong neutrophil migration by the induction of cytokine-induced neutrophil chemoattractant-2αβ (CINC-2αβ) production and L-selectin expression [85]. An experiment found that oral tributyrin visibly ameliorated inflammatory symptoms in mice. Oral tributyrin administration attenuates murine inflammatory symptoms by suppressing TNF-α, CINC-2αβ, and NO synthesis by neutrophils via HDAC and NF-κB pathway inhibition, highlighting its therapeutic potential despite its short half-life [86]. Notably, butyrate and propionate induce caspase-8/9-mediated apoptosis in both activated and quiescent neutrophils [87, 88], with mature neutrophils additionally relying on GPR109A signaling for programmed cell death [89].

**Figure 2:**
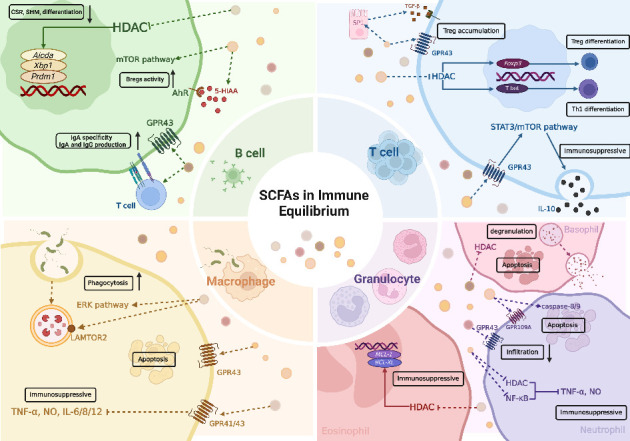
Immunomodulatory roles of SCFAs in maintaining immune equilibrium. SCFAs regulate immune cell functions via GPR41/43/109A, HDAC inhibition pathways. Key mechanisms include promoting Treg differentiation, enhancing Breg activity, and suppressing proinflammatory cytokines. SCFAs modulate apoptosis, phagocytosis, and cytokine release, balancing immune activation and tolerance through epigenetic and metabolic reprogramming. HDACs, histone deacetylases; GPR, G protein–coupled receptors; Breg, regulatory B cell; Treg, regulatory T cell; CSR, class-switch recombination; SHM, somatic hypermutation; mTOR, mammalian target of rapamycin; AhR, aryl hydrocarbon receptor; 5-HIAA, 5-hydroxyindole-3-acetic acid; TGF-β, transforming growth factor-β; Th1, T helper cell 1; IL-10, interleukin 10; EPK, eukaryotic protein kinase; NF-κB, nuclear factor kappa-B; TNF-α, tumor necrosis factor-α; NO, nitric oxide.

In allergic inflammation, SCFAs restore eosinophil homeostasis by downregulating transcription and expression of *BCL-XL* and *MCL-1* [90]. Butyrate specifically inhibits eosinophil hyperactivity by reducing CD44, CD49d, and chemokine receptor (CCR) 3, counteracting IL-5-driven adhesion and migration [90–92]. SCFAs further modulate eosinophil activity indirectly by inhibiting Th9 differentiation and impairing their immunostimulatory functions [59]. In basophils, acetate targets GPR41 to suppress Ca^2+^ influx, while propionate and butyrate boost degranulation and modulate apoptosis, accompanied by IL-13 upregulation and IL-4 downregulation [93, 94] (Figure 2).

### 3.5. Regulation of Mucosal Immunity

SCFAs critically shape mucosal immune responses, extending to systemic sites, like the airway, particularly in the context of allergic inflammation. In food allergy, by reinforcing intestinal epithelial integrity and modulating DC function, SCFAs promote oral tolerance [95]. Butyrate strengthens tight junctions (e.g., upregulating ZO-1, occludin, and claudin-1) and mucin production, reducing allergen translocation and subsequent systemic sensitization [96–98]. Concurrently, SCFAs promote IL-10-producing Tregs and Bregs, which suppress mast cell degranulation and IgE production, key drivers of anaphylaxis in food allergy [47, 94]. Clinically, *Clostridium butyricum*–derived butyrate amplifies Breg populations and IL-10 secretion during allergen-specific immunotherapy, demonstrating efficacy in allergic rhinitis and asthma [44, 45]. This is mechanistically linked to butyrate-induced mTOR activation in Bregs and upregulation of the AhR agonist 5-HIAA, which suppresses germinal center hyperactivity [46, 47]. Isobutyrate enhances gut barrier function via GPR109A, concurrently increasing the levels of beneficial metabolites, SCFAs, and 3-hydroxybutyric acid and suppressing TLR4/MyD88/NF-κB signaling pathway to exert anti-inflammatory properties [99].

In asthma, SCFAs attenuate eosinophilic inflammation through dual mechanisms, involving direct granulocyte modulation and suppression of the Th9/IL-9 axis [59, 90, 92]. Notably, SCFAs further mitigate asthma by restoring gut–lung axis homeostasis. High-fiber diets elevate circulating butyrate, which suppresses lung IL-33 release and Type 2 innate lymphoid cell (ILC2) activation [100, 101]. These findings position SCFAs as key modulators of mucosal immune equilibrium, with therapeutic potential for allergic and asthmatic disorders. Nevertheless, tissue-specific SCFA bioavailability and receptor, GPR41/43/109A, heterogeneity across mucosal sites warrant further investigation to optimize targeted therapies.

## 4. Biological Functions of SCFAs

### 4.1. SCFAs as Dual Modulators of Energy Metabolism and Systemic Regulation

SCFAs serve dual roles as metabolic substrates and systemic regulators. Collectively contributing to 10% of human energy expenditure, butyrate alone supplies 70% of intestinal energy via β-oxidation into acetyl-CoA in colonic epithelia, thereby sparing reliance on glucose and pyruvate oxidation [102–104]. Propionate predominantly fuels hepatic gluconeogenesis, while 50%–70% of acetate is metabolized by the liver and peripheral tissues for acetyl-CoA synthesis [104].

Beyond energy provision, SCFAs exhibit systemic regulatory effects. Acetate and butyrate enhance glucagon-like peptide-1 (GLP-1) and peptide YY (PYY) secretion through GPR41/43-independent mechanisms in acute settings [105], yet chronically upregulate these receptors via Ca^2+^-mediated signaling in colonic L cells [106]. Notably, DF-derived SCFAs improve glycemic control and weight management by activating intestinal gluconeogenesis (IGN). Butyrate directly stimulates IGN-associated genes via cAMP upregulation, whereas propionate engages gut–brain neural circuits to promote IGN. Notably, butyrate can also target these circuits to activate brown adipose tissue (BAT) [107, 108]. Additionally, butyrate suppresses hypothalamic neuropeptide Y-expressing orexigenic neurons [108], while acetate modulates appetite via glutamate–glutamine cycling and γ-aminobutyric acid synthesis [109]. In adipose and liver tissues, SCFAs activate mitochondrial fatty acid oxidation by constraining the activity and expression of peroxisome proliferator-activated receptor-γ (PPAR-γ) [110], while butyrate improves insulin sensitivity and increases energy expenditure in skeletal muscle and BAT via AMPK/p38 pathways and PPAR-γ coactivator-1α activation [110]. These multifaceted roles position SCFAs as therapeutic candidates for metabolic disorders like obesity and diabetes. BCFAs contribute to energy metabolism but may impair insulin sensitivity. Valerate and isovalerate are β-oxidized in hepatocytes, yielding acetyl-CoA and propionyl-CoA, yet chronic elevation associates with insulin resistance in obesity [111], underscoring the need to evaluate SCFA profiles holistically in metabolic disorders.

### 4.2. SCFAs in Shaping Gut Microbial Homeostasis

The colonic microbiota, comprising 10^14^ bacteria, relies on SCFAs to sustain mutualistic host–microbe interactions [112]. Pathological shifts in intestinal oxygen and nitrate levels favor facultative anaerobes (e.g., *E. coli*) [113–116], disrupting the original stable microflora composition, which metabolize ethanolamine and choline into harmful metabolites like acetaldehyde and trimethylamine [117, 118]. Dietary interventions boosting SCFA precursors restore symbiosis by promoting carbohydrate fermentation, acidifying luminal pH, and activating PPAR-γ in colonic epithelia. This enhances mitochondrial β-oxidation, reduces oxygen diffusion, and suppresses nitrate availability, thereby fostering dominance of obligate anaerobes (e.g., Firmicutes, Bacteroidetes) [119, 120].

### 4.3. SCFAs as Antitumor Agents

Clinical evidence links CRC progression to diminished fecal SCFA levels [121–123]. Butyrate-producing *Clostridium butyricum* suppressed Wnt/β-catenin signaling, induced tumor cell apoptosis, and attenuated high-fat diet-driven intestinal tumorigenesis in murine models [124, 125]. SCFAs exert antitumor effects heavily relying on anti-inflammatory mechanisms, counteracting chronic inflammation implicated in CRC pathogenesis [126–129]. Propionate and butyrate enhance antitumor immunity by potentiating CD8^+^ T-cell activation, particularly in microsatellite instability CRC subtypes [130]. Tumor cells evade SCFA-mediated control by downregulating GPR43/109A and SLC5A8 transporters, which reduce butyrate/propionate uptake [131, 132]. Upon internalization by cancerous cells, SCFAs upregulate proapoptotic *Bax*, *Bad*, *Bak*, and *FAS* and suppress antiapoptotic *Bcl-2* family members, which lead to caspase-3-activated mitochondrial apoptotic pathway and caspase-independent autophagy [133–136]. Furthermore, GPR43 restoration in human colorectal adenocarcinoma cells (HCT)-8 triggered p21-mediated G1/G0 cell cycle arrest upon SCFA exposure [137].

## 5. Biological Mechanisms Underlying the Regulatory Functions of SCFAs

### 5.1. SCFAs Modulate Epigenetic Modifications via HDAC Inhibition

SCFAs exert direct inhibitory effects on HDACs by competitively displacing Zn^2+^ ions at their catalytic sites, thereby suppressing enzymatic activity and elevating histone acetylation levels. This epigenetic alteration initiates downstream cascades involving chromatin remodeling enzymes and miRNA, as well as DNA and histone methylation [138–140]. Notably, HDACs exhibit structural complexity, multifunctional roles, and cell type-specific expression profiles. Their intricate interactions with diverse protein partners suggest that SCFA-mediated regulatory mechanisms extend beyond canonical pathways, warranting further investigation into unexplored molecular interfaces.

### 5.2. SCFAs Orchestrate Signaling Networks via GPR Activation

GPR41 and GPR43 exhibit differential tissue distribution and ligand selectivity. While GPR41 is ubiquitously expressed, GPR43 localizes to immune cells, adipocytes, enteroendocrine L cells, intestinal epithelial cells, and pancreatic islets β cells [141]. Among SCFAs, propionate demonstrates dual agonism for both receptors, whereas acetate preferentially activates GPR43. Butyrate and iso-butyrate exhibit higher affinity for GPR41 [141], whereas valerate and caproate display weaker activity [142]. SCFAs activate distinct GPRs, eliciting divergent signaling cascades dictated by Gα subunit specificity [141, 143]. GPR41 activation suppresses cAMP via Gi/o coupling, while GPR43 engages both Gi/o and Gq proteins, enabling cAMP inhibition or Ca^2+^/mitogen-activated protein kinase (MAPK) signaling, respectively [142].

Butyrate uniquely activates GPR109A, a Gi/o-coupled receptor expressed in adipocytes, immune cells, and intestinal epithelia, and retinal pigment epithelia [144]. Conversely, acetate and propionate stimulate that olfactory receptor (Olfr) 78, a renal and vascular receptor, in conjunction with GPR41, mediates SCFAs in blood pressure regulation [145]. Intriguingly, colonic Olfr78 activation in enteroendocrine cells regulates PYY secretion, linking SCFAs to gut hormone homeostasis and metabolic signaling [146]. This multilayered receptor interplay underscores SCFAs as versatile modulators of systemic physiology, with tissue-specific responses shaped by receptor expression patterns and ligand selectivity.

## 6. Potential Applications in Clinical Practice

The multifaceted role of SCFAs in modulating systemic homeostasis and underlying mechanisms underscores their therapeutic potential for managing chronic diseases. Below, we delineate the clinical implications of SCFAs across diverse pathologies, supported by emerging evidence from preclinical and clinical studies.

### 6.1. Diabetes Management

SCFAs exhibit promising efficacy in both T1D and T2D. In individuals with T1D and obesity/overweight, DF and carbohydrate intake positively correlate with fecal SCFA levels, particularly acetate, and promote the proliferation of SCFA-producing genera such as *Roseburia* and *Ruminococcus gnavus* [147]. Preclinical studies demonstrated that acetate and butyrate supplementation reduce diabetogenic cytokines (e.g., IL-21) and autoreactive T-cell populations in nonobese diabetic mice [148]. However, oral butyrate administration in longstanding T1D patients showed limited immunomodulatory effects, highlighting the need for optimized delivery strategies [14].

For T2D, dietary interventions enriched with SCFA precursors enhance glycemic control. Fermented milk supplementation elevated fecal acetate and attenuated systemic inflammation [149]. One randomized controlled trial (RCT) investigating diet treatment with white bean extract demonstrated improved glycemic control and reduced complications via microbiota-driven SCFA production [150]. Increased SCFA-producing bacterial diversity and abundance correlates with elevated GLP-1 secretion and improved hemoglobin A1c (HbA1c) levels [151]. Besides, amylose-rich bread consumption reduced postprandial glucose in overweight individuals, linked to elevated plasma propionate [152]. The Mediterranean diet exhibited superiority over Western diets in terms of postprandial metabolism, as evidenced by a significant increase in postprandial plasma butyrate, directly related to improved insulin sensitivity and effective enhancement of glucose metabolism [153].

### 6.2. Obesity/Overweight and Metabolic Syndrome

SCFA-producing bacteria are pivotal in combating obesity/overweight. Prebiotic intake reduces neural responses to high-calorie stimuli, potentially mediated by *Bifidobacteria* and *Lactobacillus* enrichment, despite unchanged SCFA levels [154]. Similarly, *Lactobacillus plantarum* supplementation decreased body weight and BMI in overweight individuals, accompanied by a shift from Firmicutes to *Bacteroidetes* (especially *Prevotella*) dominance [155]. Targeted colonic delivery of propionate (via inulin-propionate ester) prevented weight gain and reduced both abdominal fat accumulation and hepatocellular lipid content [156]. Furthermore, administering SCFAs via colonic injection, particularly in the distal colon region [157], enhanced fat oxidation and energy expenditure in obese men [158], while oral butyrate improved metabolic parameters in pediatric obesity [159].

### 6.3. Cardiovascular Health and Hypertension

Butyrate inversely correlates with both systolic and diastolic blood pressure [160], exerting cardioprotective effects via L-3,4-dihydroxyphenylalanine modulation and Tregs cell expansion, thereby retarding the progression of hypertension and ventricular remodeling [161]. GPR41 and Olfr78 receptors regulate renin secretion and vascular tone, serving as the foundation for blood pressure management [145]. Oral or rectal administration of propionate mitigated vascular calcification by modulating gut microbiota composition, particularly through supplementation with *Akkermansia muciniphila* [162]. Moreover, in hypertensive individuals, dietary sodium reduction intake effectively elevated circulating caproate, improving arterial compliance [163].

### 6.4. IBD

SCFAs demonstrate therapeutic efficacy and response evaluation in IBD, particularly ulcerative colitis (UC) [1, 164, 165]. Depleted *Roseburia hominis* and *Faecalibacterium prausnitzii* in UC, a characteristic alteration [166], are partially restored by localized butyrate administration, often synergizing with 5-aminosalicylic acid [13, 167–173]. Increased production of SCFAs can serve as an indicator of successful fecal microbiota transplantation (FMT) [174]. In patients who exhibited effective response to FMT and experienced sustained improvement in clinical symptoms, the remission was found to be associated with butyrate-producer recovery and *ButCoA* gene modulation. The utilization of intestinal microbiota and its metabolites, such as SCFAs, holds promise as a predictive tool to assess the response of IBD patients toward biologic interventions. Recurrence, on the other hand, was linked to Bacteroidetes and Proteobacteria abundance along with low levels of *Clostridium* clusters IV and XIVa [175]. Anti-TNF-α responders exhibited enriched Firmicutes/Bacteroidetes and reduced levels of Proteobacteria and Actinobacteria [176, 177]. Furthermore, the observed elevation in endogenous metabolites such as butyrate and deoxycholic acid is closely associated with this process, holding promise as predictive tools to assess the response of IBD patients toward biologic interventions [176]. Sodium butyrate capsules improved circadian gene expression and clinical outcomes in active UC [178], though pediatric trials showed variable efficacy [179]. This lack of efficacy may be attributed to factors such as concentration and mode of administration, and utilization of pH-sensitive encapsulation techniques can delay the release of SCFAs and improve their bioavailability [179]. In line with this, oral microcapsules containing sodium butyrate have been shown to enhance the abundance of SCFAs producers in IBD patients, such as *Lachnospiraceae* spp. and *Butyricicoccus*, thereby exerting positive effects on clinical disease activity and quality of life [180]. Notably, dietary strategies, including low-fat/high-fiber regimens, can serve as a crucial adjunctive therapy, reducing inflammation and restoring microbiota balance, as evidenced by a significant increase in Bacteroidetes and decrease in Actinobacteria, which is strongly correlated with elevated acetate levels, consequently, improving the quality of life in UC [181]. Dietary intervention involving Tetrastigma hemsleyanum polysaccharides (THP) resulted in a reversal of the intestinal ecosystem composition and increased SCFAs, accompanied by enhanced GPR41/43 signaling, thereby alleviating symptoms in DSS-induced IBD mice [182].

### 6.5. CRC Prevention

SCFAs-mediated dietary intervention is a crucial approach for the prevention of CRC. The Mediterranean diet's protective effects in chronic noncommunicable diseases can be attributed to SCFAs in safeguarding intestinal homeostasis [153, 183–186]. Disturbance in microbial composition characterized by reduced SCFA producers mediates the onset and progression of polyposis in mice. However, high-fiber diets can reverse this alteration and restore SCFA levels along with GPR109A expression, preventing carcinogenesis [53, 187]. Butyrylated starch exhibited a protective effect against the carcinogenic impacts of high-red meats diet by inhibiting O6-methyl-2-deoxyguanosine accumulation [188]. Additionally, probiotics mitigated chemotherapy-induced dysbiosis and boosted SCFA production [189].

SCFAs represent a cornerstone in the prevention and treatment of chronic diseases, including but not limited to diabetes, obesity, hypertension, IBD, and CRC. Their pleiotropic effects on metabolism, immunity, and microbiota composition underscore their translational potential. Future research should focus on optimizing SCFA delivery systems and validating clinical protocols to harness their full therapeutic promise. Personalized nutrition frameworks, integrating microbiome profiling and SCFA biomarkers, represent the next frontier in precision medicine.

## 7. Discussion and Future Perspectives

The comprehensive exploration of SCFAs in this review underscores their pivotal role as molecular orchestrators of host–microbiota crosstalk, immune equilibrium, and metabolic homeostasis. Emerging evidence positions SCFAs not merely as metabolic byproducts but as dynamic signaling molecules with pleiotropic therapeutic potential. However, translating these preclinical insights into clinical practice remains fraught with challenges, including dose- and context-dependent effects, interindividual microbiota variability, and bioavailability limitations. Addressing these gaps demands innovative strategies to harness SCFAs full therapeutic promise while navigating their biological complexity. Crucially, the efficacy of SCFA interventions is modulated by several contextual factors: (1) Baseline microbiota composition, where dysbiosis-induced depletion of SCFA producers (e.g., *Faecalibacterium* in IBD) may necessitate microbiota restoration prior to SCFA supplementation [166, 187]; (2) host disease status, as evidenced by divergent outcomes in early-stage diabetes (responsive) versus longstanding T1D (refractory) [14, 148]; (3) genetic predisposition, exemplified by polymorphisms in GPR41/43 or SLC5A8 transporters affecting ligand affinity and uptake [131, 141]; and (4) intervention modality, wherein colonic delivery (enemas/distal infusion) outperforms oral administration in UC therapy due to pH-dependent degradation and hepatic first-pass metabolism [13, 157, 179]. Precision profiling of these variables will be essential for patient stratification.

A critical innovation lies in the development of precision delivery systems tailored to optimize SCFA bioavailability and tissue specificity. Conventional oral supplementation or enema-based approaches often fail to achieve sustained luminal or systemic concentrations due to rapid metabolism or pH-dependent degradation. Advances in nanotechnology, such as pH-responsive nanoparticles, could enable targeted colonic release of SCFAs, enhancing local efficacy while minimizing off-target effects [190–193]. Furthermore, engineered probiotics expressing butyrate-synthesizing enzymes or CRISPR-edited commensals may offer a sustainable, self-renewing source of SCFAs, bypassing dietary dependency [194–196]. Such bioengineered platforms could synergize with dietary interventions to restore microbial niches in disordered conditions like IBD or CRC.

Another frontier involves integrating multiomics and machine learning to decode the “SCFA interactome.” While current studies predominantly focus on single pathways, SCFAs operate within a dynamic network of host genetics, microbial metabolism, and environmental factors. Systems biology approaches, combining metagenomics, metabolomics, and epigenomic profiling, could unravel context-specific SCFA interactions and predict patient-specific responses [197, 198]. For instance, artificial intelligence–driven models may identify microbiome signatures predictive of SCFA efficacy in autoimmune diseases or cancer immunotherapy, enabling personalized therapeutic regimens.

Moreover, the therapeutic potential of SCFA derivatives and analogs remains underexplored. Structural modifications, such as esterification or fluorination, could enhance receptor affinity or metabolic stability. For example, tributyrin, a butyrate prodrug, shows improved pharmacokinetics in preclinical models but requires optimization for human use [199, 200]. Similarly, dual-acting molecules combining SCFA moieties with existing drugs, like HDAC inhibitors, may yield synergistic effects in metabolic or oncological disorders.

Finally, large-scale longitudinal trials are imperative to validate SCFA-centric interventions across diverse populations. Current clinical data, often derived from small cohorts or short-term studies, lack the statistical power to account for microbiome heterogeneity or dietary confounders. Ultimately, bridging mechanistic insights with translational innovation, SCFA research can transition from bench to bedside, offering novel strategies to combat the global burden of chronic diseases.

## Data Availability

Data sharing is not applicable to this article as no new data were created or analyzed in this study.
